# The cavitation characteristics of aerospace high-speed centrifugal pumps with different tip clearance

**DOI:** 10.1038/s41598-024-57822-4

**Published:** 2024-03-29

**Authors:** Wen-xiong Chao, Wang-cheng Wang, Wei Zhang, Gao-yang Bai, Wei Dong

**Affiliations:** 1 Xi’an Aeronautical University, No.259 on West Second Ring Road, Xi’an, 710077 Shaanxi Province China; 2 National Joint Engineering Research Center of Special Pump System Technology, Xi’an, 710077 Shaanxi China; 3 Shaanxi Aerospace Power Research Institute, Xi’an, 710000 Shaanxi China; 4 AVIC Xinxiang Aviation Industry CO.LTD Shanghai Branch, Shanghai, 200120 China; 5 Northwest A&F University, Xi’an, 712100 Shaanxi China

**Keywords:** Gap cavitation, Different tip clearance, Cavitation characteristic, Numerical analysis, Glycol aqueous solution, Experiment, Astronomy and planetary science, Energy science and technology, Engineering

## Abstract

This study investigates the Tip Clearance Cavitation (TCC) characteristics of three different Tip Clearances (TC) (0.4, 0.6, 0.8) and five inlet negative pressure conditions Pj = (− 20–60)kPa to improve the reliability of the aerospace high-speed centrifugal pump during in-orbit operation, based on the premise of good agreement between the TC 0.6 test curve and the simulation performance curve. Under negative pressure and high-speed conditions, the variation gradient of cavitation characteristics with various inlet negative pressures is non-linear and has a sudden change, but the trend becomes stable after the inlet negative pressure drops to a certain stage. The tip clearance cavitation characteristics vary from the blade surface cavitation characteristics due to the difference in forces on both sides. This study is a proper starting point for the design of aerospace power pumps.

## Preface

During the operation of the semi-open composite impeller centrifugal pump, a certain gap known as the tip clearance^1,2^ is reserved between the impeller and the volute casing to prevent scratching and collision. Because the medium's viscosity is insufficient to keep the pressure differential between the suction and pressure surfaces of the water wing at the tip clearance, the flow bypasses the blade end face from the pressure surface and flows to the suction surface, resulting in tip clearance flow cavitation. The mainstream, secondary flow, and boundary layer of the volute casing's inner wall all have an impact on the formation and growth of the clearance flow. Despite the fact that the clearance flow area is small in comparison to the mainstream area, cavitation in the tip region is extremely complex and is frequently accompanied by tip clearance cavitation, jet shear layer cavitation, tip leakage vortex cavitation, and blade suction surface sheet cavitation.

Using water as a medium, researchers have currently analyzed and explained the characteristics of tip vortex cavitation and tip clearance cavitation. Yoshihara^3^ and Shen^4^ observed that the flow velocity inside cavitation is much lower than that in the mainstream area and that the pressure at the interface between cavitation and the mainstream area is essentially equal to the cavitation pressure at the time^5^. The effects of different turbulence models on clearance leakage cavitation calculation and the flow characteristics of clearance leakage cavitation were compared. The results depict that when the clearance is small, the shear cavitation inside the airfoil clearance is more intense, and the leakage cavitation above the airfoil is farther from the airfoil's surface. Hu Shuai, Chao Wenxiong, and colleagues^6–11^ performed a flow field analysis and experimental study of cavitation using the Mixture multiphase flow model and cavitation model for high-speed centrifugal pumps under various inlet total pressure conditions. As the cavitation coefficient decreases, the number of bubble volumes gradually occupies the entire impeller channel and the bubble distribution shifts from an asymmetric to symmetrical structure. When the effective cavitation margin reaches a certain value, the head begins to slowly decrease and then abruptly decreases. The critical cavitation margin associated with a 3% reduction in the head is investigated. The flow field and pressure transient characteristics of cavitation in high-speed fuel pumps at various flow rates were also investigated. At different flow rates, the results depict that bubbles are first generated at the leading edge of the blade. Bubbles form at the root and extended back surface of the blade as the cavitation number decreases. The Flow simulation is used to compare the cavitation margin and steam mass fraction of the current centrifugal pump and the optimized centrifugal pump and the cavitation margin of the optimized aerospace centrifugal pump is smaller than that of the current centrifugal pump.Tian Long^12^ a numerical simulation and experimental verification method are applied to research the effect of the blade tip clearance on the gas–liquid two-phase flow and cavitation characteristics of the centrifugal pump. The results show that the smaller the value of the blade tip clearance, the smaller the critical cavitation number of the model pump, where the distribution of the pressure on the blade surface and the turbulent kinetic energy in the pump of the model pump with 0.3 mm clearance value is affected by the cavitation number the least.Yang Wenjie^13^ Through numerical simulation and experimental research, showed that the working conditions required for spiral centrifugal pumps with different clearances to promote clearance cavitation were different. With the decrease of cavitation margin of this type of spiral centrifugal pump, the model pump with 1.2 mm clearance would first experience clearance cavitation. The closer the clearance of the model pump is to 1.2 mm, the greater cavitation margin is required to promote clearance cavitation.

This paper describes a low-temperature semi-open impeller high-speed centrifugal pump with an ethylene glycol water solution as the working medium that is used in specific types of space suits. The space system has higher expectations and demands for small volume, high-speed centrifugal pumps, which can easily cause cavitation inside the pump, resulting in decreased hydraulic performance, continuous high-frequency and high-pressure impacts of bubbles on the metal surface of the overcurrent components, causing metal damage and shortening the service life. As a result, the need for cavitation prevention in high-speed pumps is becoming increasingly pressing. Traditional civil or industrial centrifugal pump analysis and research have primarily focused on clean water and low-speed states. The cavitation phenomenon of high-speed operation and multi-component non-Newtonian ethylene glycol water solution are thoroughly discussed in this paper. There is currently little research data on various tip clearance cavitation characteristics for ethylene glycol water solution in high-speed centrifugal pumps. As a result, it is critical to investigate the various tip clearance cavitation characteristics of special media for miniaturized and high-speed centrifugal pumps used in space temperature control systems.

## Model and test analysis

### Test high-speed pump

**Figure 1:**
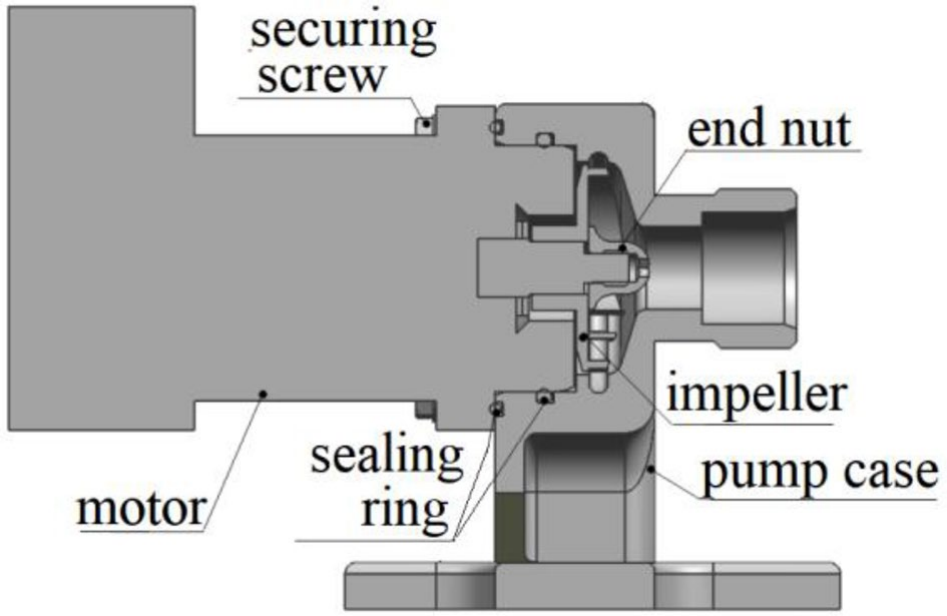
Structure of centrifugal pump.

The parameters of the study object are as follows: the rated flow *Q*_v_ = 0.000111 m^3^/s, the boost value *Δ*P = 170 kPa, the rated speed n = 9400 r/min, the specific speed n_s_ = 32, the blades are two-dimensional cylindrical, the medium is glycol aqueous solution, and the impellers are 4 * 4 semi-open compound type. The geometrical dimensions of the pump are shown in Table 1. Clearance between pump body and blade top TC (0.4, 0.6, 0.8). The tip clearance TC size is adjusted using stainless steel adjustment screws and centrifugal pump structure in Fig. 1.

**Table 1:** Results of geometric parameters.

Parameters	Value/mm
Width of blade inlet	6.4
Width of blade outlet	3
Diameter of impeller inlet	14
Diameter of impeller outlet	35
Axial width of volute	3.2

### Verification of hydraulic characteristics test

**Figure 2:**
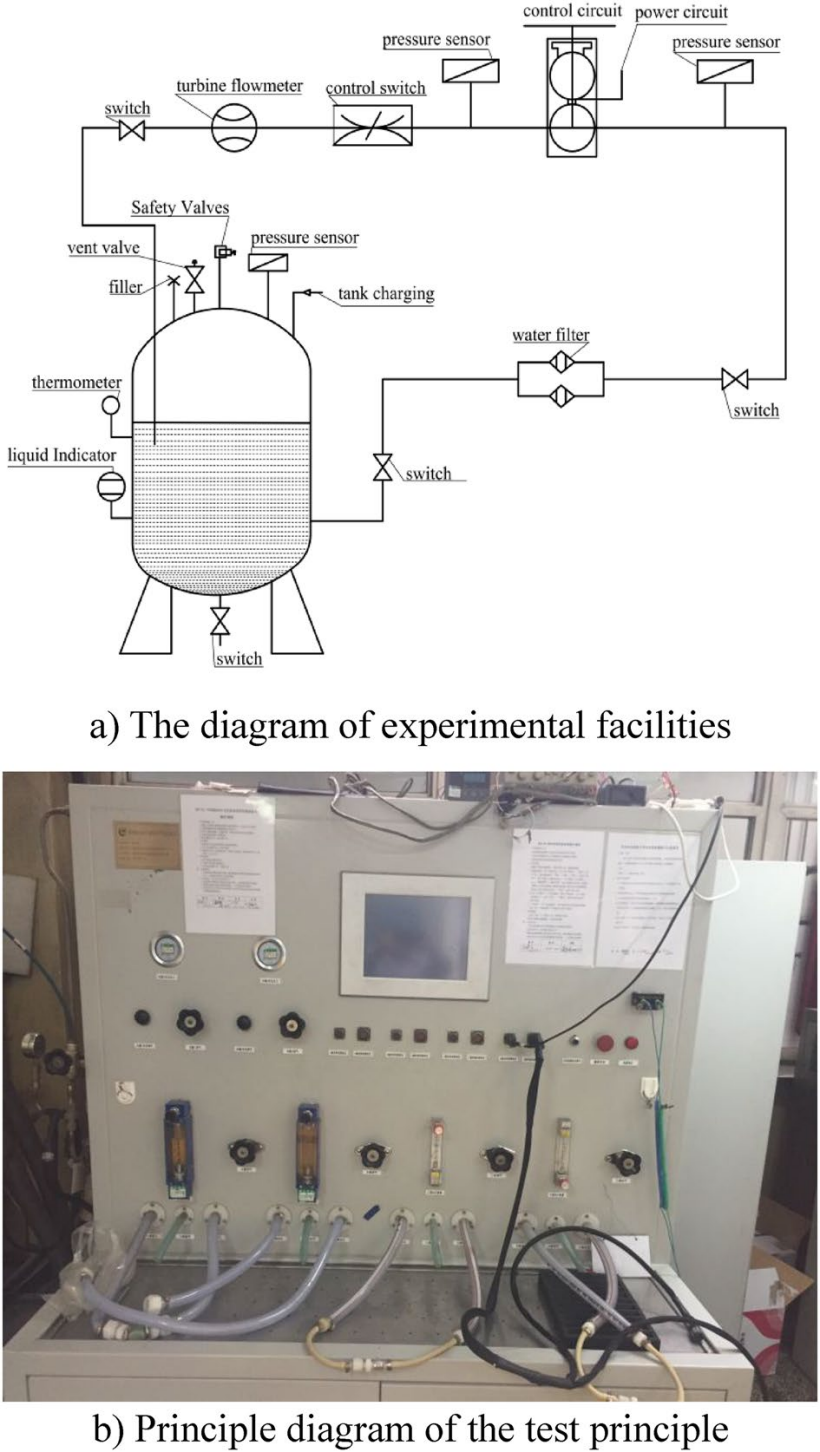
The centrifugal pump experimental facilities.

The cavitation test was performed on a hydraulic test platform using a four-blade composite centrifugal pump in accordance with GB/T3216-2005. The density of the ethylene glycol aqueous solution is 1030 kg/m^3^, the temperature is 7.6 °C, and the flow rate *Q*_V_ = 400 L/h. The initial inlet pressure P_j_ = 0 kPa. The pump's inlet pressure was progressively reduced by adjusting the inlet valve until the reduced value of inlet and outlet pressure difference was 3% of the rated value. The critical cavitation margin was achieved at this point. The cavitation test results were compared to the numerical simulation findings to ensure that the numerical simulation is accurate and the test setup is shown in Fig. 2.

In order to ensure the smoothness and finish of the surface of the impeller and volute, the impeller is finished on the basis of surface roughness of 1.6 μm. The volute was anodized on the basis of surface roughness of 1.6 μm. The roughness of the internal surface after treatment is very small.

**Figure 3:**
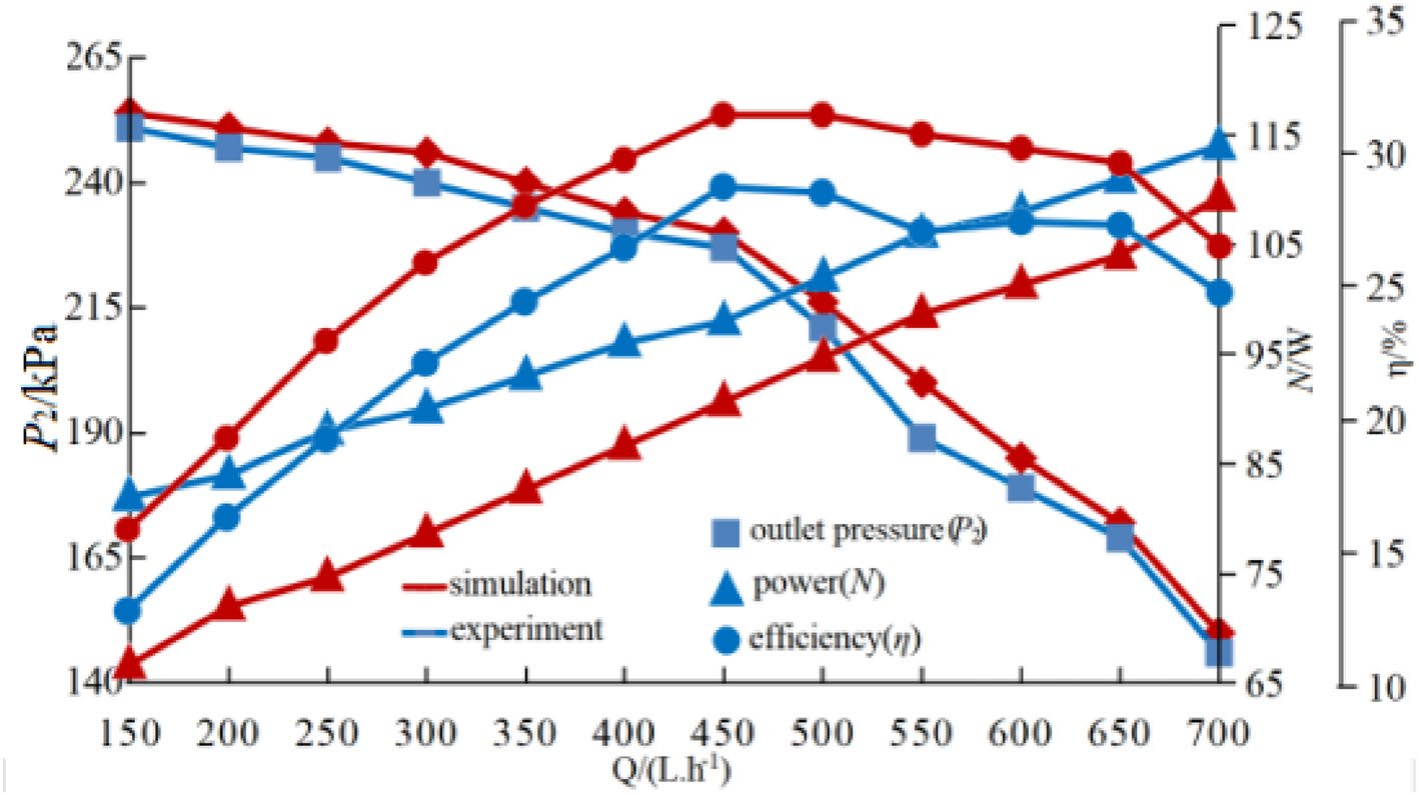
Performance curves of various working conditions.

Figure 3 depicts the difference in performance between the experimental value and the performance parameter anticipated by the numerical simulation of the cylindrical backswept compound impeller blade top TC = 0.6 and the design operation point in the absence of cavitation. The errors between the head value and the test value are all less than 5%, showing that the numerical calculation simulation can simulate the internal flow field of the centrifugal pump at the design operation point and the numerical simulation results presented in this paper are accurate. Although the power error does not surpass 10%, the error is still significant because the mechanical friction loss of various bearings and friction pairs during centrifugal pump operation is not taken into account in the numerical simulation process.

### Cavitation performance at various flows

**Figure 4:**
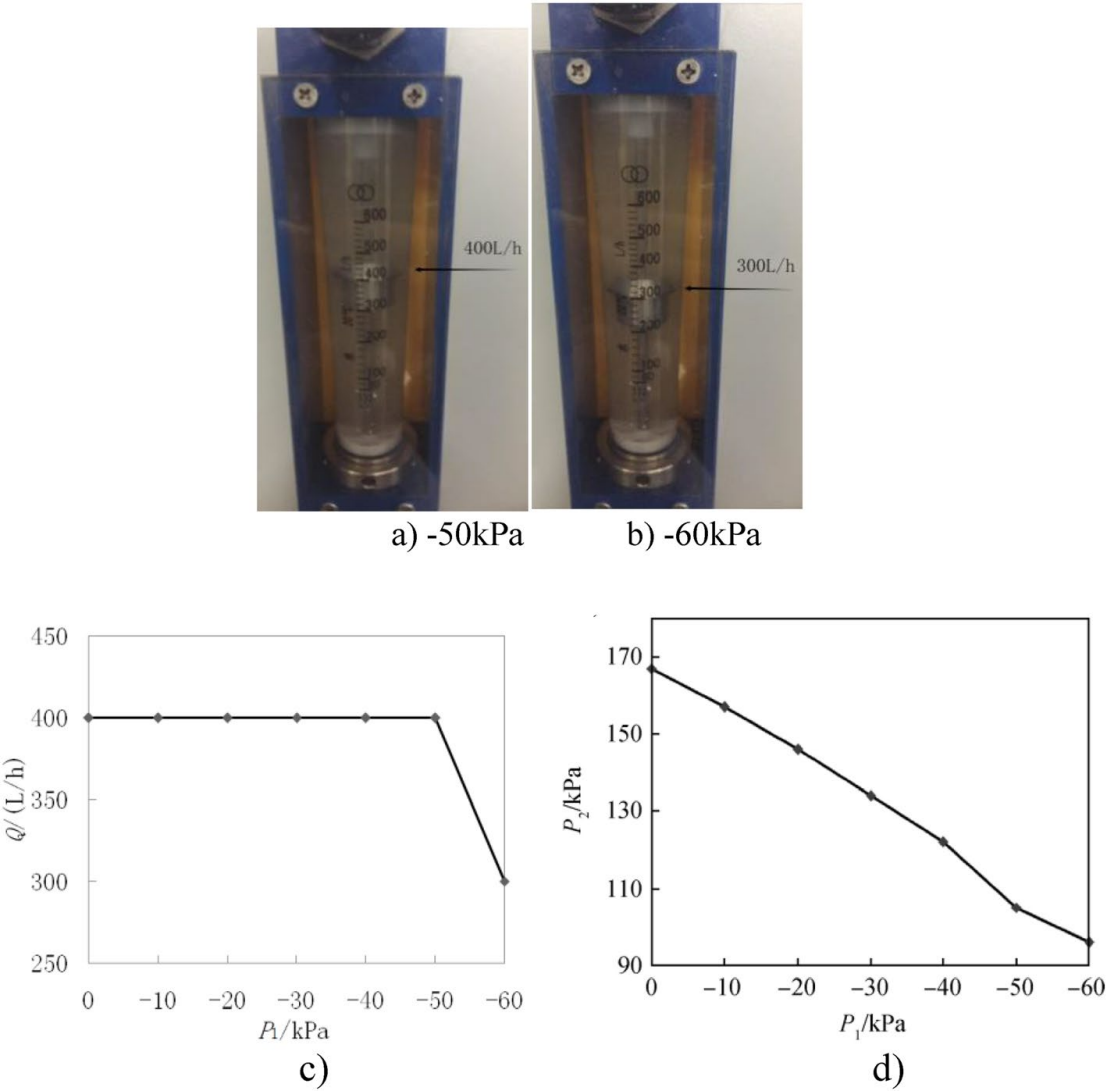
The change of outlet flow rate with inlet negative pressure.

While the pump flow rate is still at the design value of Q_v_ = 0.000111 m^3^/s = 400 L/h and the performance hasn't suddenly dropped, Fig. 4a depicts the early stages of cavitation with bubbles already emerging at the bottom of the flowmeter. The abrupt decrease in pump flow rate to Q = 0.000083 m^3^/s = 300 L/h in Fig. 4b indicates that cavitation has achieved the required level of 3%.

Figure 4c depicts the cavitation characteristic curve and the experimental performance value graph of a high-speed centrifugal pump with a cylindrical backswept compound impeller operating at various flow rates. The pressure difference between the inlet and output is approximately 3% of the rated pressure difference when the inlet negative pressure P_j_ = − 50 kPa. The crucial cavitation outlet pressure at this moment is the pump outlet pressure P_2_ = 105 kPa.

Figure 4d depicts the inlet negative pressure is (0–(−50))kPa, the outlet flow rate of the pump is maintained at 400 L/h, and the pressure difference between the inlet and outlet of the pump is 155 kpa, which is close to the limit range of 3% of the rated pressure difference. At this time, the outlet pressure of the pump is 105 kPa, which is the critical cavitation outlet pressure. The inlet pressure is far less than the minimum inlet pressure of 10 kPa when the pump is working normally, which proves that the design of the centrifugal pump is reasonable, and the pump can fully adapt to the anti-cavitation performance requirements of the centrifugal pump.

## Calculation method and meshing

### Governing equation

The ethylene glycol aqueous solution is moving in a complex, unstable three-dimensional turbulent flow with the Reynolds time-averaged N–S equations as follows:

1 $$ \left\{ {\begin{array}{*{20}l} {\nabla \bullet u = 0} \hfill \\ {\rho \frac{du}{{dt}} = \rho F - \nabla P + \mu \nabla^{2} u} \hfill \\ \end{array} } \right. $$

where $\nabla$ is the vector operator in the Cartesian coordinate system, u is the velocity vector of the fluid; P is the fluid pressure; F is the force vector per unit mass; $\rho ,\;\mu$ are the density and molecular viscosity of the fluid, respectively.

### Cavitation phase transition model

Based on the concept of the two-phase flow model, Pumplinx numerical simulation software uses the entire cavitation model "Cavitation" to function. The "Cavitation" model introduces the idea of mixing density, which integrates the non-condensable gas, evaporation condensation processes, and the compressibility of liquids^14–16^. It also solves the dynamic process of the phase transition of the cavitation bubble using the bubble dynamics Rayleigh–Plesset equation. Furthermore, a huge number of engineering projects have put this approach to the test and proven it.

The Singhal^17^ model and the N–S equation were combined, and the RNG k–ε turbulence model was adopted tosolve the equation. The relationship between the density changing rates of density and vapor phase volume fraction were obtained by fusing the Singhal model with the continuity equation as follows:

2 $$ \frac{{D\rho_{{\text{m}}} }}{{D{\text{t}}}}{ = } - (\rho_{{\text{l}}} - \rho_{\upsilon } )\frac{{D\alpha_{\upsilon } }}{{D{\text{t}}}} $$

where vapor volume fraction *α*_v_ is related to cavitation bubble number density and cavitation bubble diameter *R*_B_:

3 $$ \alpha_{\upsilon } {\text{ = n}}(\tfrac{4}{3}\pi R_{B}^{3} ) $$

Singhal model is based on the Rayleigh–Plesset bubble dynamics equation:

4 $$ \frac{P\upsilon - P\infty }{{\rho_{l} }} = R_{B} \frac{{{\text{d}}^{2} R_{B} }}{{dt^{2} }} + \frac{3}{2}(\frac{{{\text{d}}R_{B} }}{dt})^{2} + 4\frac{\mu }{R}\frac{{{\text{d}}R_{B} }}{dt} + 2\frac{\sigma }{{\rho R_{B} }} $$

The expression for the phase transition rate is obtained by neglecting the viscosity and surface tension effects and combining the continuity equation of each phase:

5 $$ R = \frac{{3\alpha_{\upsilon } }}{{R_{B} }}\frac{{\rho_{\upsilon } \rho_{l} }}{{\rho_{m} }}(\frac{2}{3}\frac{{p_{B} - p}}{{\rho_{l} }})^{1/2} $$

where *P*_B_ is the saturation vapor pressure, σ is the surface tension coefficient, and *α*_v_ is the vapor phase volume fraction.

The phase change rate is connected to the density of the vapor phase, the density of the liquid, and the density of the mixture in the simulation of internal flow cavitation and cavitation erosion of centrifugal pumps. The Singhal model accounts for the effects of turbulence and non-condensable gas. The Singhal vapor phase mass fraction transport equation is as follows:

6 $$ \frac{\partial }{\partial t}\left( {\varphi_{\upsilon } \rho_{\upsilon } } \right) + \nabla \bullet \left( {\varphi_{\upsilon } \rho_{\upsilon } \overline{{v_{\upsilon } }} } \right) = {\text{R}}_{e} - R_{c} $$

where $\varphi_{\upsilon }$ is vapor volume fraction and $\overline{{v_{\upsilon } }}$ is the average vapor phase velocity; *R*e and *R*c are the phase change rates of vaporization and condensation, respectively, and the expressions are as followed:

7 $$ \begin{gathered} \left\{ {\begin{array}{*{20}c} {{\text{Re}} = 3\frac{{\rho_{\upsilon } \rho_{1} }}{{\rho_{m} }}.\frac{{\varphi_{\upsilon } \left( {1 - \varphi_{\upsilon } } \right)}}{{R_{b} }}\sqrt {\frac{2}{3}.\frac{{p_{\upsilon } - p}}{{\rho_{1} }}} ;\quad if\; p < P_{\upsilon } } \\ {{\text{Re}} = 3\frac{{\rho_{\upsilon } \rho_{1} }}{{\rho_{m} }}.\frac{{\varphi_{\upsilon } \left( {1 - \varphi_{\upsilon } } \right)}}{{R_{b} }}\sqrt {\frac{2}{3}.\frac{{p - p_{\upsilon } }}{{\rho_{1} }}} ;\quad if\; p > P_{\upsilon } } \\ \end{array} } \right. \hfill \\ R_{B} = \left\{ {3\varphi_{\upsilon } /\left[ {4\pi n\left( {1 - \varphi_{\upsilon } } \right)} \right]} \right\}^{1/3} \hfill \\ \end{gathered} $$

### Calculation method

The SIMPLE algorithm is used, as well as the full cavitation model "Cavitation." The ethylene glycol aqueous solution is the first phase, and the ethylene glycol aqueous solution bubble is the second. 1.41 kPa is the saturated vapor pressure of ethylene glycol aqueous solution.

The centrifugal pump impeller speed is 9400 r/min and the given volute outlet volume flow rate *Q*_v_ = 0.000111 m^3^/s are used in the calculation. The NPSH of the pump is changed during the numerical calculation by gradually decreasing the inlet pressure from 0 Pa to control the cavitation degree in the pump. The liquid phase's initial volume fraction ratio is one, while the cavitation bubble phase's is 0.

### Meshing

The accuracy of the numerical simulation results is determined by the quality of the fluid domain grid. PumpLinx's CFD simulation solution employs the finite volume method for CFD simulation solution and that is the unstructured grid. PumpLinx grid generator uses a proprietary CAB geometric equirectangular height adaptive binary tree Cartesian coordinate algorithm as well as a more regular Cartesian hexahedral mesh that can generate body-fitted grid near wall surfaces. By continuously splitting the grid, the CAB algorithm can automatically adjust the grid size to fit the geometric surfaces and geometric boundary lines. When generating grids for automatically encrypted resolution of the geometry of complex details, an adaptive algorithm was also used. The proprietary grid generation algorithm has a lower grid number than the tetrahedral mesh at the same accuracy level.

**Figure 5:**
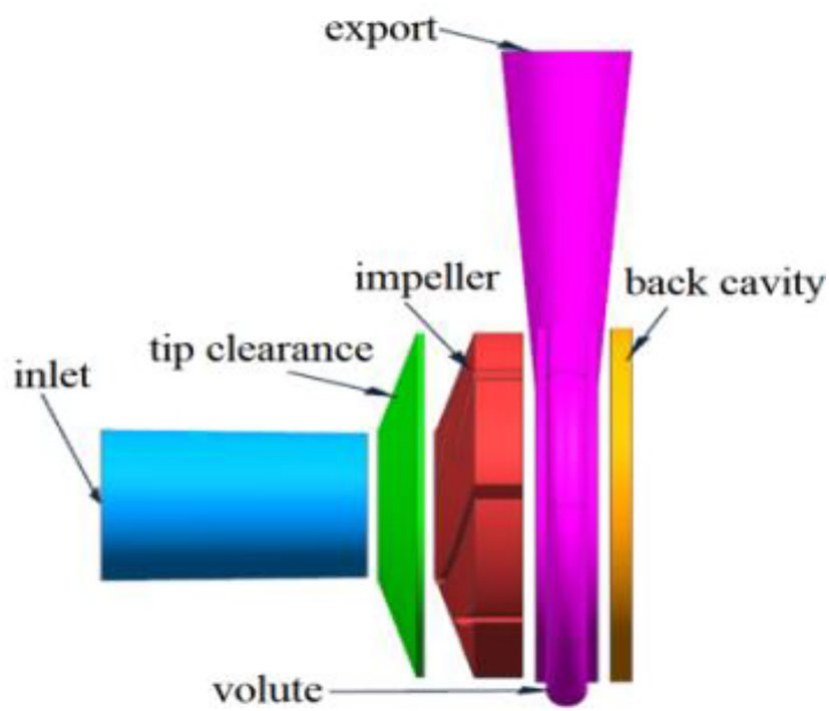
Computational domain of Centrifugal Pump.

**Figure 6:**
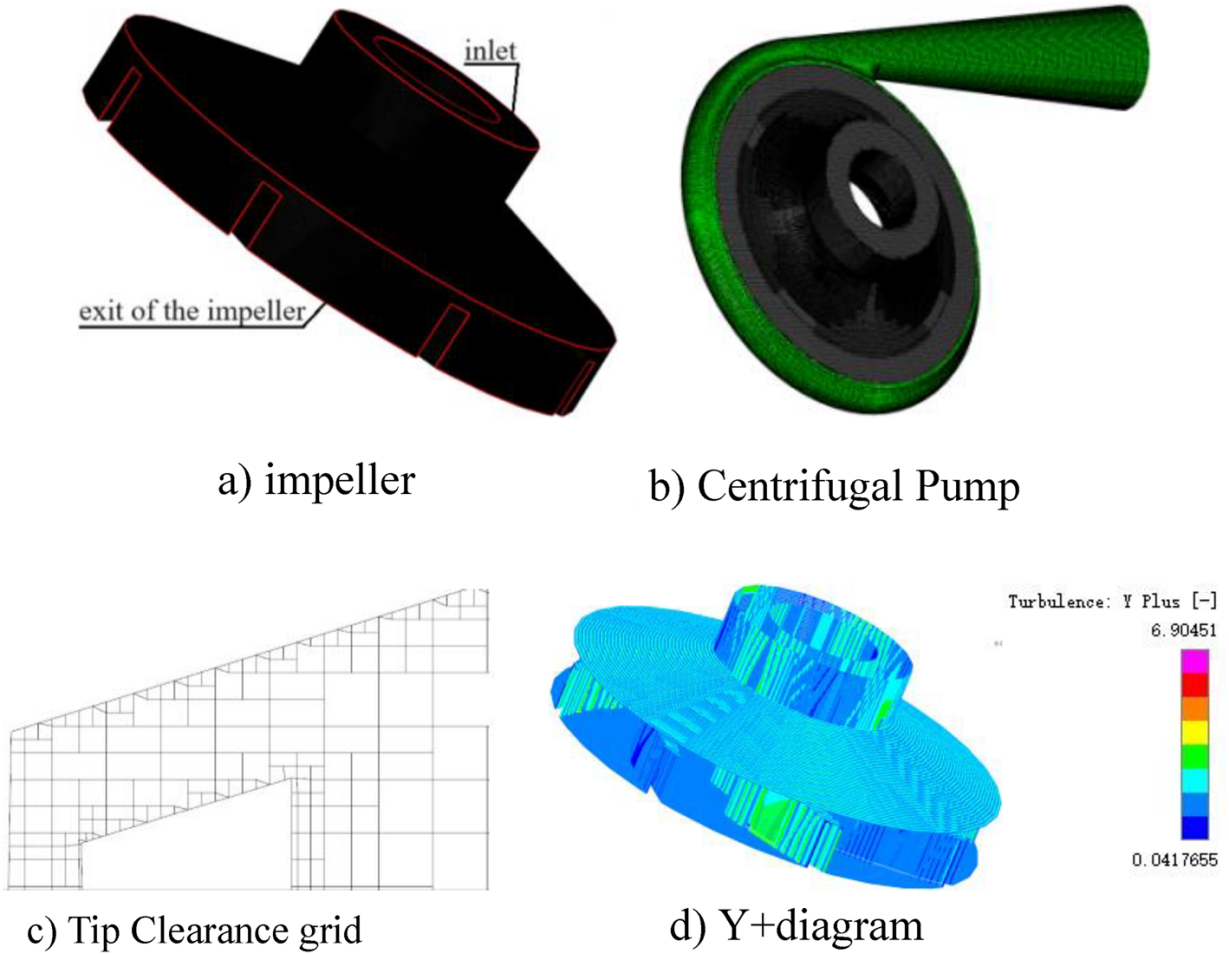
Computation grid diagrams.

Figure 5 depicts the three-dimensional flow channel of the flow passage component, Where the TC(0.4,0.6,0.8) and Fig. 6 depicts the grid diagram of the impeller, where the computational domain consists of the impeller, the volute, and the tip clearance layer of the impeller. Because the focus of this research is on the cavitation characteristics of high-speed centrifugal pumps, the grid topology, number of grids, and grid aspect ratio are all carefully controlled to ensure a relatively smaller size difference between adjacent grid nodes and more accurate numerical simulation results. Figure 6dThe first wall layer y +  = 0.0417 ~ 6.9045, the average wall layer y +  = 3.4731. The verification of grid independence, the total number of grids in the computing domain are finally estimated to be approximately 2.6 million, taking into account the computer capability and efficiency, Verification of grid independence as shown in Table 2.

**Table 2:** Verification of grid independence.

The number of grids/ten thousand	Pressure gain *ΔP*/Pa
≈150	173,590
≈204	173,371
≈230	173,326
≈260	173,219
≈290	173,215
≈320	172,317

## Cavitation calculation results and analysis

### Analysis of cavitation on the intermediate cross section at different inlet negative pressures

Figure 8 depicts the internal bubble volume distribution inside the pump at site A is φ24 for three different TC values when the inlet negative pressure is P_j_ = − 20 kPa, − 30 kPa, − 40 kPa, − 50 kPa and − 60 kPa at the designed flow rate.The position dimensions of A and B are shown in Fig. 10. Outside of the φ24 impeller's range, no cavitation occurred. The cavitation areas for the three TC values follow a consistent pattern: cavitation areas exhibit rotating cavitation characteristics as the impeller rotates at high speeds which are primarily concentrated on the blade suction surface(because the suction surface pressure is lower than the pressure surface), and the cavitation intensity is greatest near the impeller blade leading edge (because the leading edge pressure is lower than the rest of the position). The cavitation areas change between the three different TC values, and the cavitation impact is stronger when the inlet negative pressure is greater than the TC value.

Figure 8 shows that the combination of P_j_ = − 60 kPa/TC 0.4 which has the strongest cavitation intensity with volume fractions almost 1, and the largest cavitation area appearing approximately circular. On the other hand, the combination of P_j_ = − 20 kPa/TC 0.8 which has the weakest cavitation intensity with volume fractions below 0.5, and the smallest cavitation area appearing approximately rectangular. This is mainly due to the increasing pressure on imports.

**Figure 7:**
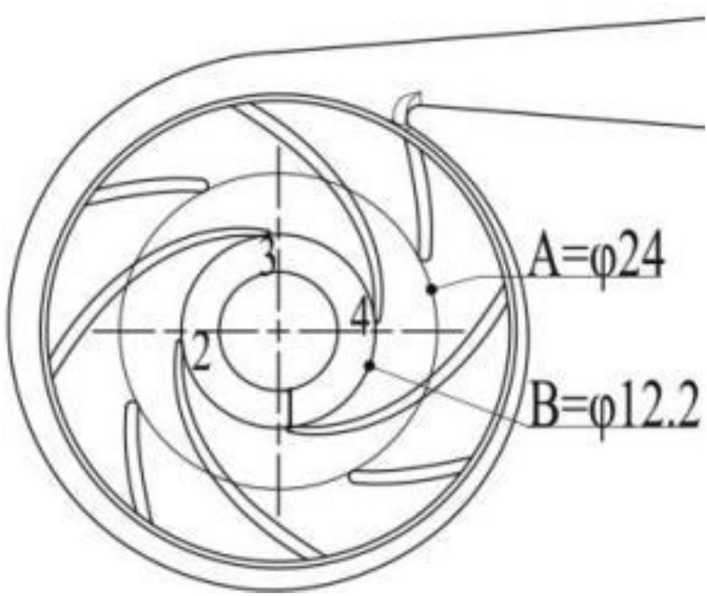
Labeled graph.

During the process of P_j_ = − 60 kPa to P_j_ = − 20 kPa, the gradient of the cavitation volume fraction changes in the order of TC 0.8 > TC 0.6 > TC 0.4, owing to the fact that the flow state is more susceptible to change with greater TC values under low flow rate and high-speed conditions influenced by the inlet pressure. The cavitation strength steadily lowers when the inlet negative pressure varies from P_j_ = − 60 kPa to P_j_ = − 20 kPa, and the volume fraction value decreases from below 0.8 to less than 0.65. Furthermore, the cavitation level fluctuates regionally, with the cavitation area near location A is φ24 gradually diminishing to the rectangular area tangent to the circle with location B is φ12.2, where the cavitation areas of grades d, e, and f decrease most significantly, The interval values of a–j are shown in K in Fig. 7.

Taking TC0.6 as an example, the cavitation intensity value gradually decreases from A to J, cavitation first occurs at the A value, the cavitation area gradually expands inward to the hub, but also to the impeller outlet, as the impeller radius increases, the pressure gradually increases and reaches the bubble bursting pressure, squeezes the bubble, and cavitation is in the φ24 circular region. When the g-value cavitation region extends to the next blade working surface, the bubble develops from the tip to the middle region of the blade to 25% chord length, but when it encounters a higher pressure area at the working surface, the cavitation extension head begins to contract and form a crescent. Due to the combined pressure from the F and D values, a hook-shaped region is formed at the E value.

**Figure 8:**
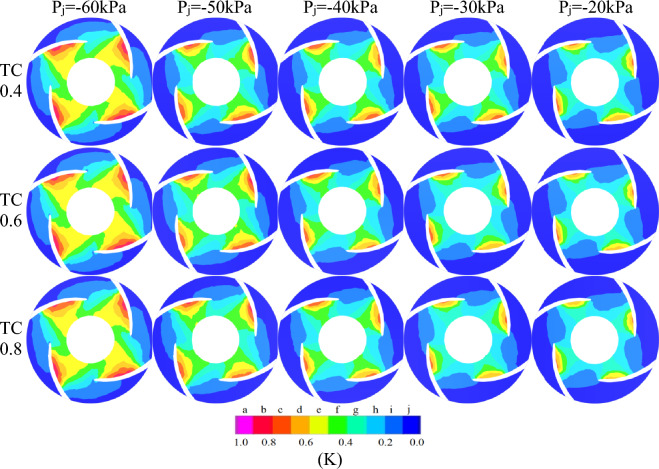
The cavitation bubble volume distribution diagrams of the pumps midsections.

As shown in Fig. 8, the asymmetry of the volute casing of the centrifugal pump's volute casing generates an unequal distribution of pressure and velocity at the connection between the impeller and the casing, and the blades are staggered in a 4 * 4 arrangement. Due to the non-orthogonality of the volute casing, the cavitation regions on the four blades are not exactly the same. As the inlet pressure increases, the gradient of the change in cavitation intensity at position 1 is the fastest, shifting from the strongest to the weakest as the inlet pressure increases,The position of 1–4 are shown in Fig. 7.

### Comparison of tip cavitation between different inlet negative pressures

**Figure 9:**
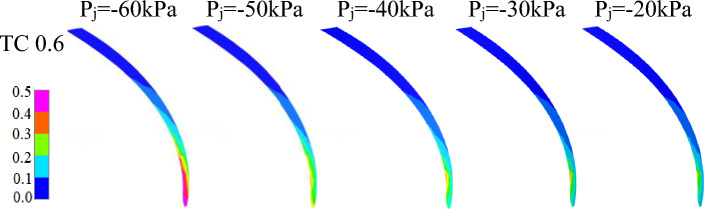
Vapour distribution in tip surface of different inlet negative pressure.

A comparison of Figs. 8 and 9 reveals that in contrast to the region with the greatest cavitation at position in Fig. 8, the region with the greatest cavitation in Fig. 9 occurs more towards the blade tip inlet. The main reason for this is that the pressure on the pressure side of the blade is higher than that on the suction side, causing some fluid to flow around the tip edge and enter the axial gap between the casing and the blade, resulting in leakage flow at the tip clearance of the blade, eventually returning towards the impeller inlet.

As shown in Fig. 9, the tip clearance cavitation strength gradually decreases from the back side of the blade to the working surface and is influenced by the high-speed rotation of the impeller. The tip cavitation has a curved arc distribution, with the arc radius increasing gradually as the cavitation strength diminishes.

**Figure 10:**
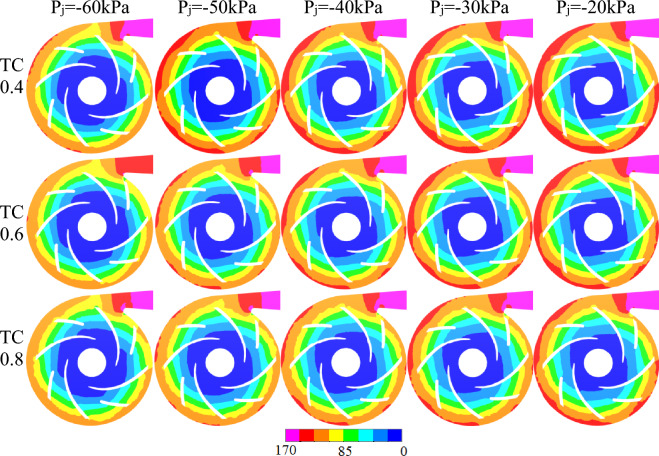
Pressure distribution on middle stream surface of impeller.

Figure 10 shows the inlet negative pressure of the centrifugal pump under the designed flow rate (Pj = (− 20 kPa; − 30 kPa; − 40 kPa; − 50 kPa; − 60 kPa)) Pressure distribution diagram in the middle section of the impeller at different times. From the inlet to the outlet of the pump, the pressure gradually increases, and the pressure change law of the blade suction surface and the pressure surface gradually increases along the direction of the flow of the medium in the impeller. The increase of blade surface pressure reflects the work characteristics of the blade working surface, that is, the work of the blade increases with the increase of the blade contact liquid area, and the pressure increases along the liquid flow direction. The low pressure area appears at the inlet of the pump (where cavitation occurs), but because of the asymmetry of the volute of the centrifugal pump, the pressure distribution in the pump is asymmetric. With the increase of negative pressure at the pump inlet, the distribution of bubbles on the blade surface gradually increased, and gradually expanded from the low pressure area of the blade to the flow channel, the low pressure area in the pump increased, and the high pressure area began to decrease. When the bubble diffuses with the liquid flow to the outlet, it will collapse and erode the blade due to the increase of pressure, which will cause the decline of the characteristic curve outside the pump.

### Analysis of bubble inside pump under unequal inlet negative pressure

**Figure 11:**
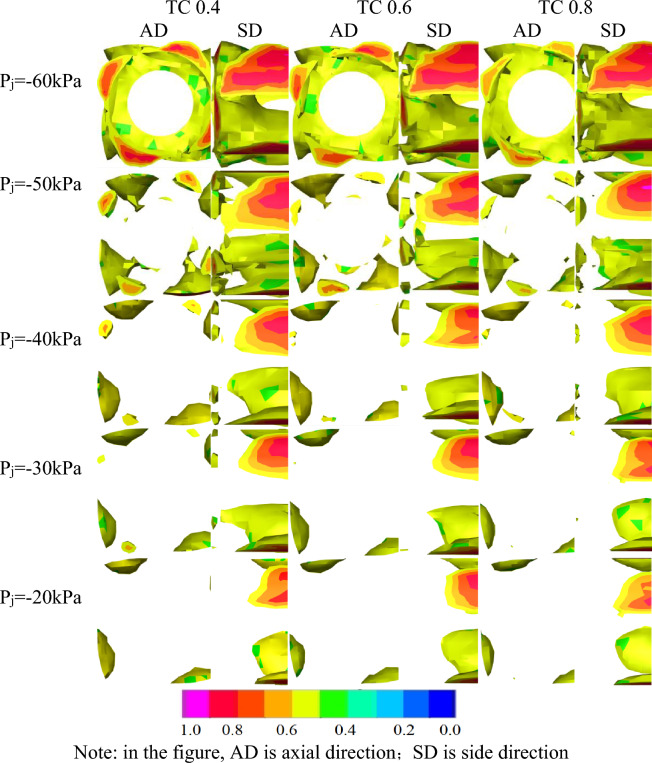
Different TC of gas phase volume fraction distribution graph under different inlet negative pressure.

As shown in Fig. 11, the evolution of bubble inside the impeller was observed at three different TC values and five different inlet negative pressure values. In the axial and lateral views of the impeller, bubble areas with a volume fraction larger than or equal to 0.5 were removed, revealing the following characteristics:

1. When the pump's inlet negative pressure decreases, so does the dispersion and volume of the bubble gradually decrease. There is a difference in the volume change of the bubble due to the asymmetric distribution of pressure and velocity at the dynamic and static coupling of the impeller caused by the asymmetry of the centrifugal pump volute and the 4 * 4 staggered layout of the blade. There is a certain difference in the volume change of the bubble with the most severe area located at position 1,The position of 1–4 are shown in Fig. 7.
2. The axial image at P_j_ = − 0.06 MPa which depicts the highest number of bubbles, causing complete or severe cavitation and obstructing liquid outflow. The bubble intensity decreases from level 0.9 to 0.7, and the bubble volume gradually decreases, although the amplitude is minimal. In the lateral perspective, there is a bubble with a level 1 intensity throughout the height of the blade. As the bubble spreads towards the outlet with the flow, the increased pressure erodes the blade, resulting in a drop in the exterior characteristic curve of the high-speed centrifugal pump.
  The influence of viscosity on the bubble reduces as the TC of the tip clearance increases, and the dominant effect of pressure difference becomes more prominent. When the clearance is tiny, the combined impact of viscosity and pressure differential drive the leakage flow through the clearance. As a result, when the clearance is large, the bubble has consistent stability due to the combined action of the pressure change on the working and non-working surfaces of the blade, as opposed to the combined effects of viscosity and pressure difference in a small clearance, resulting in a more stable cavitation characteristic.
3. The volume of bubbles reduces in magnitude from P_j_ = − 0.06 MPa to P_j_ = − 0.05 MPa. The bubbles which previously occupied the entire inlet, now occupy only the space in front of the suction surface of the blade. The number of bubbles is relatively uniform and appears in blocks, distributed according to the four blades at the suction surface's front edge. The bubbles have an intensity of less than 0.7. The volume of bubbles at the blade's tip is significantly smaller than that at the blade's root, and they appear in sporadic blocks in the lateral view. The bubble intensity is at level 1 in a small area at the blade's root. The pump's performance is usually normal at this time.
4. The bubble volume drops from P_j_ = − 0.05 MPa to P_j_ = − 0.04 MPa, and the bubble occupying the blade suction surface leading edge space decreases to nearly half of its size at P_j_ = − 0.05 MPa. The cavitation strength level is locally present in the 0.7 range, but is typically below 0.6. The side view graph depicts that at P_j_ = − 0.05 MPa, the bubble volume in the tip direction shrinks to roughly one-third of its original size and only appears irregularly. TC 0.4 > TC 0.8, and the cavitation strength level is less than 0.9.
5. As observed in the axial image, the volume of the bubble reduced from P_j_ = − 0.04 MPa to P_j_ = − 0.03 MPa. When viewed in conjunction with the lateral view, it can be observed that the sporadic block bubbles at the blade inlet reduced to very small sporadic points at TC 0.6 and TC 0.8, while the cavitation intensity level still exists in a limited area of 0.9.
6. From P_j_ = − 0.03 MPa to P_j_ = − 0.02 MPa, the bubbles at the inlet disappear on the axial view, while the volume of bubbles in the blade's height direction decreases on the lateral view.

## Conclusion

This article analyzes the cavitation characteristics of ethylene glycol aqueous solution in a high-speed centrifugal pump of the space thermal control system under different inlet negative pressures, and comes to the following conclusions:

1. As the tip clearance TC value increases, the effect of fluid viscosity reduces in comparison to the small clearance, and the dominant role of pressure difference becomes prominent. The leakage flow through a small clearance is driven by both viscosity and pressure differential. As a result, at high clearances, the pressure changes on the blade working and non-working surfaces have consistent stability whereas in small clearances, the combined effect of viscosity and pressure differential have inconsistent stability. The cavitation features are more constant, and the strength and volume of the bubbles diminish. The effect of fluid viscosity reduces as the tip clearance TC value increases, the effect of fluid viscosity decreases compared to that in the small clearance, and the dominant role of pressure difference becomes prominent. In small clearances, both viscosity and pressure difference drive the leakage flow through the clearance. Therefore, in large clearances, the pressure changes on the blade working and non-working surfaces have consistent stability, whereas in small clearances, the combined effect of viscosity and pressure differential have inconsistent stability. The cavitation characteristics are more stable and the cavitation intensity and bubble volume decrease.
2. The most significant reduction in bubble volume intensity and number occurred from P_j_ = − 0.06 MPa to P_j_ = − 0.05 MPa, followed by a less pronounced change from P_j_ = − 0.05 MPa to P_j_ = − 0.04 MPa.
3. When compared to the blade, the area with severe cavitation in the blade clearance is more slanted towards the blade tip inlet. The cavitation intensity gradually weakens from the back of the blade to the working surface, and the tip cavitation area is distributed in a circular arc shape. Moreover, the radius of the circular arc increases gradually.
4. The combination of P_j_ = − 60 kPa/TC 0.4 has the highest cavitation intensity, whereas the combination of P_j_ = − 20 kPa/TC 0.8 has the least.

## Data Availability

Some of data generated or analysed during this study are included in this published article. The current study are not publicly available due Reasons for privacy of experimental data and equipmen,but are available from the corresponding author on reasonable request.
